# An injury profile of basketball players in Accra, Ghana

**DOI:** 10.4102/sajp.v75i1.467

**Published:** 2019-03-27

**Authors:** Jonathan Quartey, Setordzor F. Davor, Samuel K. Kwakye

**Affiliations:** 1 Department of Physiotherapy, School of Biomedical and Allied Health Sciences, College of Health Sciences, University of Ghana, Accra, Ghana; 2 School of Medicine and Dentistry, College of Health Sciences, University of Ghana, Accra, Ghana; 3 West Africa Football Academy, Accra, Ghana

## Abstract

**Background:**

Basketball is played in Ghana at amateur and professional levels. The demands (cardiovascular, musculoskeletal and metabolic capacity) of the game place players at risk of sustaining injuries. There is a dearth of evidence of injuries sustained during basketball games in Ghana.

**Objectives:**

The aim of this study was to determine the injury profile of Ghanaian basketball players.

**Method:**

This observational cross-sectional study was conducted at the Lebanon House and Prisons courts during the 2013 Greater Accra Basketball league for Division 1 and 2 male basketball teams. Injuries were recorded according to body part injured, causes of injury, player’s ability to return to play following an injury, type of injury and treatment received using a standardised injury report form and data capturing form. Twenty-eight league competitions and 28 training sessions were observed. Data were analysed using a *Z*-test for two proportions.

**Results:**

Seventy-five injuries were recorded and the injury incidence was 0.190 and 0.084 per 100 participants during competition and training, respectively. Tackling attempts (42.67%) were the most common causes, followed by others (30.67%), which were dribbling, landing from a jump, sudden stops and jumping. Sprain (28%) was the most common injury. Knee injuries (21.33%) were more common than ankle injuries (17.33%). Out of the total injuries recorded, 85.33% did not receive any treatment.

**Conclusion:**

Knee injuries were the most common and most injuries did not receive treatment. It is therefore important to educate basketball players and coaches on injury prevention measures as well as developing regular exercise programmes to help minimise their occurrence.

**Clinical implications:**

Basketball injuries appear to be common so the outcomes of this study may provide prophylactic interventions and more focussed treatments regimens for basketball injury incidences in Accra.

## Introduction

Basketball is a team sport played by five players in two opposing teams following a set of rules with the aim of scoring in the opponents’ basket and preventing them from scoring (International Basketball Federation 2012). Apart from national basketball competitions, basketball players in Ghana are engaged in various domestic basketball competitions. Such competitions include community dunk, sprite ball, junior ball, inter-high school competitions and tertiary-level institution games. Currently, 18 teams are registered with the Greater Accra Basketball Association, which is a member of the Ghana Basketball Association. Of these teams, eight are in male Division 1, six belong to the women’s division and four are in male Division 2.

Traditionally, basketball was considered a non-contact sport, but now increasing body contact suggests that it has evolved into a semi-contact sport (Mckay & Cook 2010). The dynamic nature of basketball, characterised by repetitive jumping, loading, landing, running, sharp and sudden changes in direction as well as sideways cutting manoeuvres (Jones, Louw & Grimmer 2000), predispose the musculoskeletal system to injuries. Most basketball injuries are orthopaedic in nature and commonly include ligament sprains, musculotendinous strains and overuse injuries, including stress fractures (Newman & Newberg 2010). Ankle and knee injuries are by far the most common injuries sustained during participation in basketball games (Jones et al. 2000). A study conducted among elite female basketball athletes revealed that the ankle is the most common site of injury (McCarthy, Voos & Nguyen 2013). Their study further elaborated that ankle sprain (47.8% of players), hand injury (20.8%), patellar tendinitis (17.0%), ACL injury (15.0%), meniscus injury (10.5%), stress fracture (7.3%) and concussion (7.1%) were the most common injuries reported (McCarthy, Voos & Nguyen 2013).

In the analysis of injury type, Drakos and colleagues (2010) showed that sprains were the most common (27.8%) injury among athletes in the National Basketball Association. Collision, falls, jumping and landing on a player’s foot, being struck by another player, pivoting, running and cutting manoeuvres are the mechanisms of injury (Burnham et al. 2010).

Some acute basketball injuries sustained during participation can be treated by protection, rest, ice application, compression and elevation – the PRICE principle. According to Adebayo, Akinbo and Odebiyi (2008), in their study the commonest immediate treatment rendered was cryotherapy followed by massage and bandaging. Physiotherapists can further contribute to the reduction of basketball-related injuries by developing and implementing injury prevention programmes for players.

In developed countries there is increasing documentation of injuries for various sporting disciplines, including basketball, which has led to efforts being made to reduce or prevent the occurrence of sport-related injuries. However, there seems to be a paucity of literature about basketball injuries in Ghana. This invariably makes it difficult to develop proper injury prevention strategies to help minimise or prevent the occurrence of such injuries or create awareness of such injuries among players, which will help them achieve maximum performance.

Information on the causes of injury, injured body part, type of injury, treatment received and player’s ability to return to play following an injury will be valuable for team managers, medical team members and players in adopting appropriate injury management and preventive measures. Thus the aim of this study was to identify the pattern of injuries among Ghanaian basketball players in Accra, Ghana.

## Methods

This observational cross-sectional study involved the 273 male players of Division 1 and 2 basketball teams in Accra. These teams participated in the 2013 Greater Accra Basketball league season and training sessions. The study was conducted at the Lebanon House and Prisons courts, where the league competitions of the 2013 Greater Accra Basketball league season took place, as well as at the various training centres of the various teams in Accra.

Information letters explaining the purpose of the study and seeking permission to conduct the study were sent to the Ghana Basketball Association and Greater Accra Basketball Association. Contact with the various team managers and teams was made, the purpose of the study was explained to them and permission to conduct the study was sought. Written informed consent was sought and obtained from all participating teams.

An injury was defined as being a basketball accident with a sudden, direct cause or onset, which required at least minimum (medical) care, including, for example, ice or tape, and which caused the injured player to miss out on at least one training or game session (Verhagen et al. 2004).

A standardised injury report form (Appendix 1) adopted from Adebayo et al. (2008) and an additional data capturing form (Appendix 2) designed by the authors was used to record the data during the 2013 league competitions and training sessions.

The adopted standardised injury report form consisted of three tables. The body part injured, causes of injuries, player’s ability to return to play following an injury, number of injuries and the total percentage of injuries were captured in Tables 1, 2 and 3. The data capturing form consisted of two tables. One table captured the type of injury sustained and the other captured initial treatment following injuries.

Following the occurrence of an injury, contact was made with the injured player and the injury confirmed by the authors. Twenty-eight league competitions (Appendix 3) and twenty-eight training sessions were observed, during which injuries were recorded with respect to the body part(s) injured, cause(s) of injury, player’s ability to return to play following an injury, type of injuries sustained and treatment received.

The reported injury incidence for this study during competition and training was per 100 participants based on a denominator of 273 participants’ exposure.

## Data analysis

Data collected were summarised as counts, frequencies and percentages. Differences in the proportions of body parts injured, causes of injury, player’s ability to return to play following an injury, type of injury and treatment received at training sessions and competitions were determined using the *Z*-test for two proportions. The difference in upper and lower limb, knee and ankle injuries were also compared using the Z-test of proportions. All tests were two-tailed and interpreted as significant at a *p*-value less than 0.05. All statistical tests were conducted using STATA version 11.0.

### Ethical considerations

Approval to conduct the study was sought and obtained from the Ethics and Protocol Review Committee of the School of Biomedical and Allied Health Sciences, University of Ghana (SAHS-ET10303011/AA/13A/2012-2013).

## Results

### Incidence of injury during competition and training

A total of 52 injuries for competition and 23 for training were recorded. The injury incidence per 273 participants’ exposures during competition was 0.190 and 0.084 for training.

### Body part injured during competition and training

There was a non-significant (*p* = 0.248) higher proportion of head injury during competition (7.14%) compared to that of training sessions (0.0%) (Table 1). There was a higher percentage of hand injuries occurring during training than during competition (*p* = 0.039). A total of 16 (21.33%) injuries occurred to the knee and 13 (17.33%) to the ankle for all injuries recorded (competition and training) as shown in Table 2. The difference between the frequency of knee and ankle injuries was not significant (*p* = 0.532).

**TABLE 1:** Body part injured during competition and training.

Body part	Competition session		Training session		*Z*	*p*
	Number (*n*)	Percentage	Number (*n*)	Percentage		
Head	4	7.69	0	0.00	1.159	0.248
Eye	7	13.46	0	0.00	1.816	0.069
Face and mandible	5	9.62	1	4.55	0.402	0.688
Arm	3	5.77	0	0.00	0.967	0.335
Hand	5	9.62	5	22.73	2.059	0.039*
Wrist	2	3.85	1	4.55	0.356	0.721
Groin	0	0.00	0	0.00	0.000	-
Hip	2	3.85	0	0.00	0.861	0.440
Thigh	2	3.85	3	13.64	1.547	0.122
Knee	10	19.23	6	27.27	1.126	0.206
Leg	2	3.85	0	0.00	0.861	0.388
Ankle	8	15.38	5	22.73	0.772	0.440
Foot	1	1.92	2	9.09	1.723	0.084
Spine and back	1	1.92	0	0.00	0.573	0.566

**Total**	**52**	**100.00**	**23**	**104.55**	**-**	**-**

* , *p* < 0.05.

**TABLE 2:** Comparison of upper and lower extremities injury.

Body part	Number (*n*)	Percentage	*Z*	*p*
Lower extremity	41	54.67	0.978	0.327
Knee	16	21.33	0.624	0.532
Ankle	13	17.33		
Upper extremity	16	21.33	**-**	**-**

### Causes of injury during competition and training

A high proportion of injuries was caused by tackling attempts during competition (50%) as compared to training (26.09%), but the difference was not significant (*p* = 0.131), as shown in Table 3. The proportion of injury during competition and training resulting from dribbling, landing from a jump, sudden stops and jumping (considered as ‘others’) showed a significant difference (*p =* 0.007) as compared to individual fall, goal post fall, interference of fans, tackling attempts and accidental contact. The highest cause of injury was tackling attempts (32, 42.67%), followed by others (23, 30.67%) and accidental contact (11, 14.67%) for the overall causes of injuries recorded.

**TABLE 3:** Causes of injury during competition and training.

Causes	Competition session		Training session		*Z*	*p*
	Number (*n*)	Percentage (%)	Number (*n*)	Percentage (%)		
Individual fall	5	9.62	4	17.39	0.954	0.339
Goal post fall	0	0.00	0	0.00	0.000	-
Interference of fans	0	0.00	0	0.00	0.000	-
Tackling attempts	26	50.00	6	26.09	1.511	0.131
Accidental contact	10	19.23	1	4.35	1.937	0.053
Others (specify)	11	21.15	12	52.17	2.680	0.007*

**Total**	**52**	**100.00**	**23**	**100.00**	**-**	**-**

* , *p* < 0.05.

### Player’s ability to return to play following an injury during competition and training

A total of 36 (69.23%) and 20 (86.96%) players returned to play immediately with some signs (pain and/or limping) of injury during competition and training sessions, respectively. More players returned immediately to playing with some signs (pain and/or limping) of injury as a result of training than during competition (*p* = 0.030) (Table 4). Of the total injuries recorded among participants, four (7.69%) and one (4.35%) did not return to play after treatment during competition and training sessions, respectively.

**TABLE 4:** Player’s level of recovery after injury during competition and training.

Remarks	Competition session		Training session		*Z*	*p*
	Number (*n*)	Percentage (%)	Number (*n*)	Percentage (%)		
Player returned immediately without sign of injury	5	9.62	2	8.70	0.901	0.123
Player returned immediately with some signs of injury	36	69.23	20	86.96	2.165	0.030*
Player returned after some minutes without signs of injury	7	13.46	0	0.00	1.627	0.103
Player did not return to play after treatment	4	7.69	1	4.35	0.523	0.601

**Total**	**52**	**100.00**	**23**	**100.00**	**-**	**-**

* , *p* < 0.05.

### Type of injury sustained during competition and training

Sprains (21, 28%) accounted for the highest injury rate recorded, followed by contusions and bruises (20, 26.67%) and lacerations (13, 17.33%) (Table 5). The other injuries included facial nerve injury and re-injuries. More sprains occurred during training sessions than during competitions (*p* = 0.009).

**TABLE 5:** Type of injury sustained during competition and training.

Type of injury	Competition session		Training session		*Z*	*p*
	Number (*n*)	Percentage (%)	Number (*n*)	Percentage (%)		
Sprain	10	19.23	11	47.83	2.582	0.009*
Strain	3	5.77	3	13.04	1.021	0.307
Dislocation	0	0.00	0	0.00	0.000	-
Fracture	0	0.00	0	0.00	0.000	-
Subluxation	0	0.00	0	0.00	0.000	-
Concussion	0	0.00	1	4.35	1.523	0.128
Laceration	11	21.15	2	8.70	1.265	0.103
Abrasion	7	13.46	1	4.35	1.186	0.236
Contusion (bruise)	17	32.69	3	13.04	1.726	0.084
Muscle cramp	1	1.92	2	8.70	1.402	0.161
Others (specify)	3	5.77	0	0.00	1.092	0.278

**Total**	**52**	**100**	**23**	**100**	**-**	**-**

* , *p* < 0.05.

### Treatment received following injury during competition and training

Forty-two (80.77%) and twenty-two (95.65%) of the injuries that occurred during competition and training, respectively, did not receive any treatment as shown in Table 6. There was no significant difference (*p* = 0.097) between injuries that received treatment and those that did not during competition and training. Of the injuries that received treatment, cryotherapy was the most administered treatment during competition and training.

**TABLE 6:** Treatment received during competition and training.

Initial treatment	Competition session		Training session		*Z*	*p*
	Number (*n*)	Percentage (%)	Number (*n*)	Percentage (%)		
Cryotherapy or ice	6	11.54	0	0.00	1.621	0.105
Massage	0	0.00	0	0.00	0.000	-
CPR or breathing	0	0.00	0	0.00	0.000	-
Bandaging or taping	0	0.00	0	0.00	0.000	-
Exercise	1	1.92	0	0.00	0.624	0.532
Bleeding control	3	5.77	1	4.35	0.177	0.859
No treatment	42	80.77	22	95.65	1.659	0.097

**Total**	**52**	**100.00**	**23**	**100.00**	**-**	**-**

## Discussion

The incidence of injury was higher during competition than in training. A study by Rechel and colleagues in 2008 corroborates this finding. An Injury Surveillance Project conducted by the National Athletic Trainers’ Association in 1995–1997 reported injury rates ranging from 1.5 to 5.0 times higher in competition compared with training in 10 sports studied, including basketball. Higher rates of injury during competition may be a result of increased play intensity, increased legal and illegal physical contact, and increased exposure to high-risk activities.

Of all body parts injured, the knee was the most frequently injured part. This finding is similar to reports by some authors (Kostopoulos & Dimitrios 2010; Louw, Grimmer & Vaughan 2003; Owoeye et al. 2012) but contrary to other study reports, which indicate the ankle as the most frequently injured body part (Adebayo et al. 2008; Borowski et al. 2008).

There were more injuries to the lower extremity than to the upper extremity. This finding is similar to previous findings by Dick et al. (2007) and Kostopoulos and Dimitrios (2010). The occurrence of more injuries to the lower extremity may be attributed to the characteristic nature of basketball, involving repetitive jumping, loading, landing, running, sharp and sudden changes in direction and sideways cutting manoeuvres, which occur in the lower extremity, predisposing it to sustaining more injuries than the upper extremity. The knee being the most commonly injured joint of the lower extremity may be attributed to improper landing technique adopted by players from jumping, as well as repetitive jumping. This may also be linked to the observation that the majority of the players do not use knee braces.

Tackling attempts, dribbling, landing from a jump, sudden stops and jumping were the most common causes of injuries recorded in our study. Tackling attempts accounted for the highest cause of injuries recorded. This seems to corroborate findings by Louw et al. (2003), Burnham et al. (2010) and Owoeye et al. (2012), who indicated that jumping and landing was the most common cause of injury, but Louw et al. (2003) cited falling as the second most common mechanism for reported injuries.

The desire of players to win the ball from their opponents or prevent them from making a shot in order to acquire more points may have contributed to tackling attempts representing the highest cause of injuries. This calls for adherence to the rules of the game, fair play, insistence on the use of protective equipment by all players and implementation of punitive measures by match officials when necessary during basketball games.

Of the total injuries recorded for competition and training, 36 (69.23%) players returned to competition immediately with some signs of injury. This is suspected to be a result of the lack of proper and adequate treatment for the majority of injuries that occurred. Players were not followed up to determine how their injuries would have affected their participation and performance in games in terms of the number of day(s) lost after injuries.

Sprain was the most common injury, followed by contusion and laceration. Similarly, other authors described sprain as the most common injury (Adebayo et al. 2008; Borowski et al. 2008; Dick et al. 2007; Kostopoulos & Dimitrios 2010). The occurrence of contusion and laceration as the next most commonly occurring injuries may be attributed to the fact that tackling attempts were the most common cause of injuries in our study (Adebayo et al. 2008). Of the total injuries recorded for competition and training, 64 (85.33%) did not receive any treatment. This finding is not different from that obtained by Louw et al. (2003), which was that 63.4% of injuries sustained were not treated. For injuries receiving treatment, cryotherapy was the most frequent treatment administered, as was the case in Adebayo et al. (2008) and Owoeye et al. (2012), who also reported cryotherapy as the most commonly administered treatment following an injury. The frequent use of cryotherapy may be a result of the important role it plays in the management of acute symptoms and signs when used effectively and appropriately.

Most injuries did not receive any meaningful treatment because of the obvious lack of a physiotherapist, doctor or first aid personnel on each team. Because of the lack of treatment following an injury, most players returned to play almost immediately either limping or with signs of injury (pain).

These findings suggest the need to educate both coaches and players on the importance of proper treatment by professionals following an injury as well as developing injury prevention programmes for teams in order to minimise the occurrence of injuries among basketball players.

## Conclusion

Tackling attempts were the highest cause of injury and the knee was the most affected region of the body. Most of the injuries did not receive any meaningful treatment, resulting in players returning to play with some signs of injury. Officials must therefore ensure fair play and encourage the use of protective equipment as a means of minimising the occurrence of injuries. It is important to educate players and coaches on the need to administer first aid following an injury. Injury prevention measures should be developed to help minimise the occurrence of injuries. Further studies should be undertaken to involve players of all basketball teams in Ghana to help develop a national injury profile.

## Figures and Tables

**FIGURE 1-A1:**
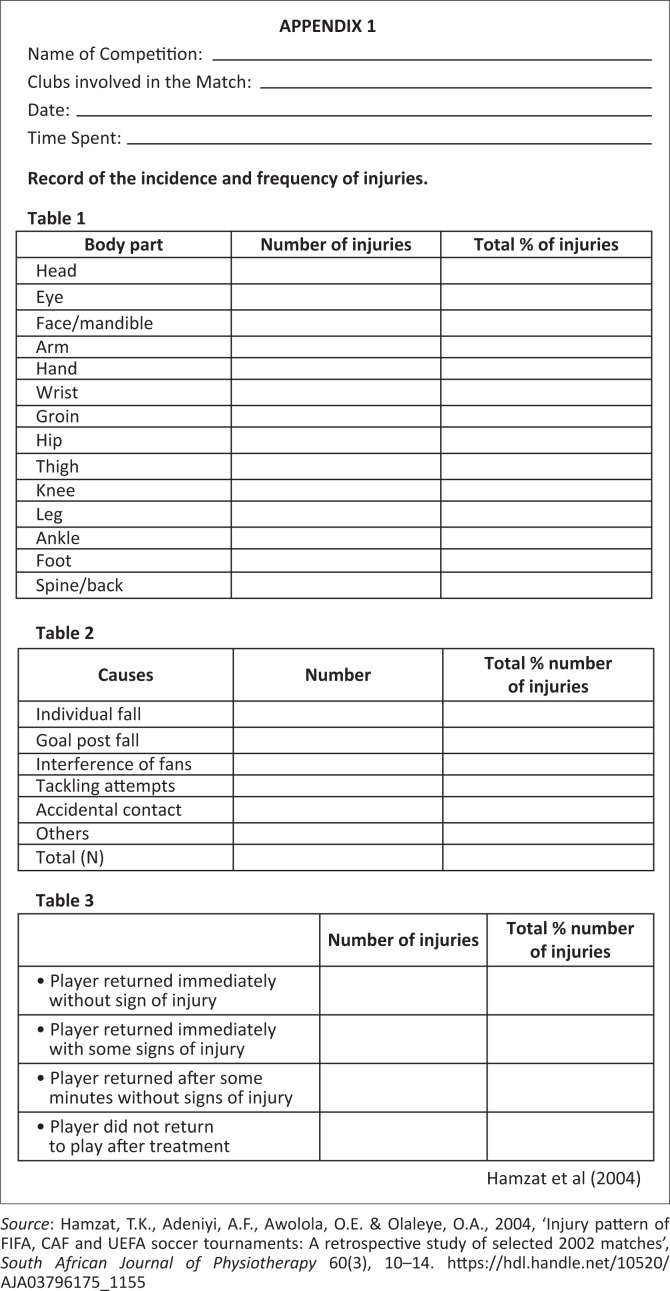
Injury report form.

**FIGURE 1-A3:**
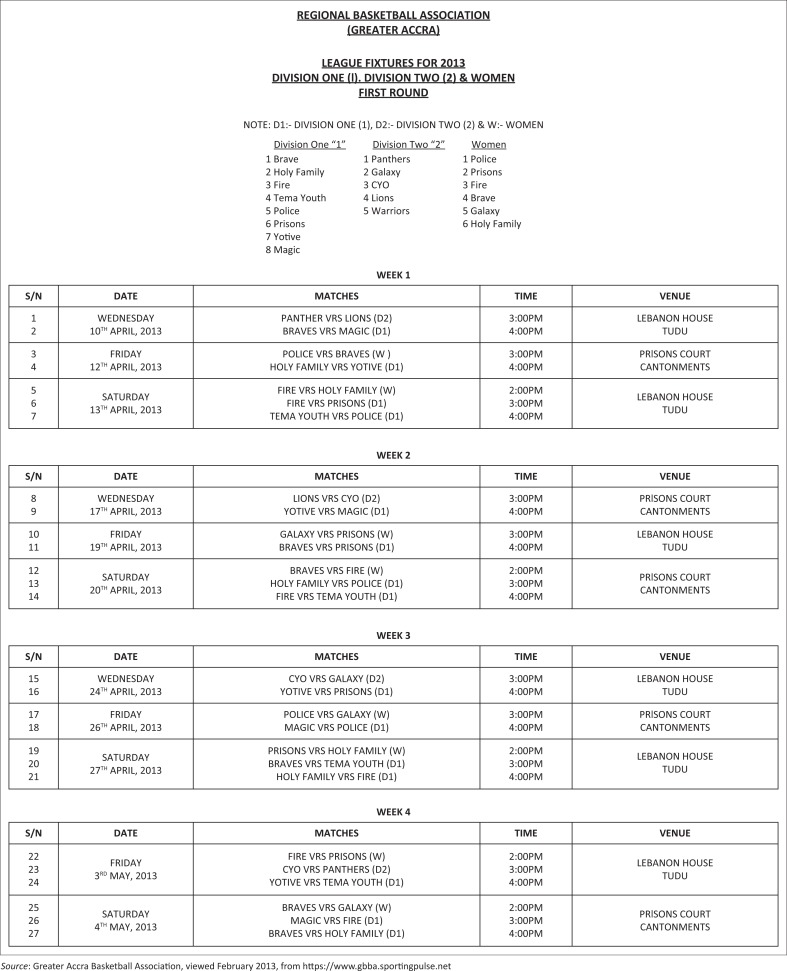

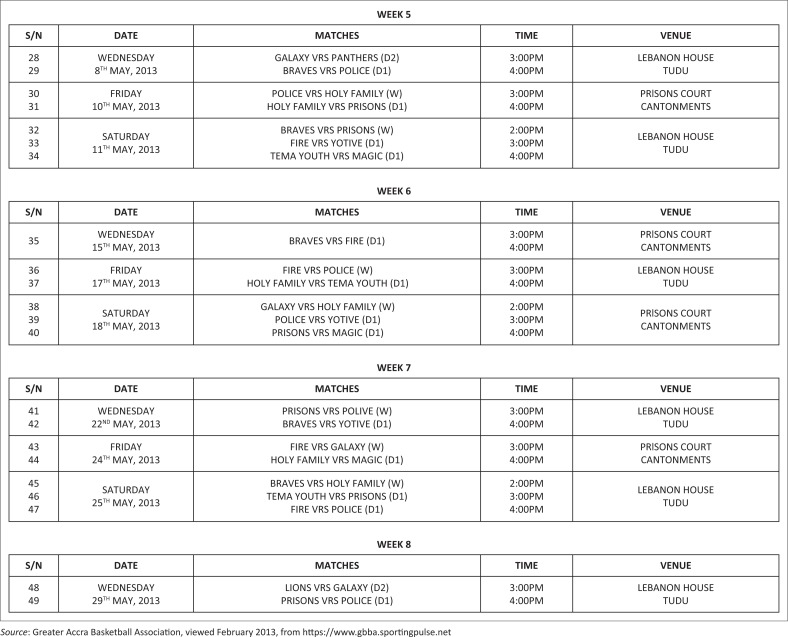
League fixtures.

## Appendix 2

### Data capturing form

**TABLE 1-A2:** Injury information.

Type of injury	Number
Sprain	
Strain	
Dislocation	
Fracture	
Subluxation	
Concussion	
Laceration	
Abrasion	
Contusion (bruise)	
Muscle cramp	
Others (specify)	

**TABLE 2-A2:** Initial treatment information.

Initial treatment	Number
Cryotherapy/ice	
Massage	
CPR/breathing	
Bandaging/taping	
Exercise	
Bleeding control	
No treatment given	
